# Genetic diversity among eight *Dendrolimus* species in Eurasia (Lepidoptera: Lasiocampidae) inferred from mitochondrial COI and COII, and nuclear ITS2 markers

**DOI:** 10.1186/s12863-016-0463-5

**Published:** 2016-12-22

**Authors:** Alexander Kononov, Kirill Ustyantsev, Baode Wang, Victor C. Mastro, Victor Fet, Alexander Blinov, Yuri Baranchikov

**Affiliations:** 1 Institute of Cytology and Genetics, the Siberian Branch of the Russian Academy of Science, 10 Prospekt Lavrentyeva, Novosibirsk, 630090 Russia; 2 USDA-APHIS-PPQ CPHST, Otis Laboratory, Building 1398, Otis Air National Guard Base, Buzzards Bay, MA 02542 USA; 3 Department of Biological Sciences, Marshall University, 1601 5th Avenue, Huntington, WV 25755 USA; 4 V.N. Sukachev Institute of Forest, the Siberian Branch of the Russian Academy of Science, 50/28 Akademgorodok, Krasnoyarsk, 660036 Russia

**Keywords:** Dendrolimus, Phylogeny, Pests, Interspecific hybridization, Divergence

## Abstract

**Background:**

Moths of genus *Dendrolimus* (Lepidoptera: Lasiocampidae) are among the major pests of coniferous forests worldwide. Taxonomy and nomenclature of this genus are not entirely established, and there are many species with a controversial taxonomic position. We present a comparative evolutionary analysis of the most economically important *Dendrolimus* species in Eurasia.

**Results:**

Our analysis was based on the nucleotide sequences of COI and COII mitochondrial genes and ITS2 spacer of nuclear ribosomal genes. All known sequences were extracted from GenBank. Additional 112 new sequences were identified for 28 specimens of *D. sibiricus*, *D. pini*, and *D. superans* from five regions of Siberia and the Russian Far East to be able to compare the disparate data from all previous studies. In total, 528 sequences were used in phylogenetic analysis. Two clusters of closely related species in *Dendrolimus* were found. The first cluster includes *D. pini*, *D. sibiricus*, and *D. superans*; and the second, *D. spectabilis*, *D. punctatus*, and *D. tabulaeformis*. Species *D. houi* and *D. kikuchii* appear to be the most basal in the genus.

**Conclusion:**

Genetic difference among the second cluster species is very low in contrast to the first cluster species. Phylogenetic position *D. tabulaeformis* as a subspecies was supported. It was found that *D. sibiricus* recently separated from *D. superans*. Integration of *D. sibiricus* mitochondrial DNA sequences and the spread of this species to the west of Eurasia have been established as the cause of the unjustified allocation of a new species: *D. kilmez*. Our study further clarifies taxonomic problems in the genus and gives more complete information on the genetic structure of *D. pini*, *D. sibiricus*, and *D. superans*.

**Electronic supplementary material:**

The online version of this article (doi:10.1186/s12863-016-0463-5) contains supplementary material, which is available to authorized users.

## Background

Moths of the genus *Dendrolimu*s (Lepidoptera: Lasiocampidae) are among the main pests of coniferous forests worldwide [1]. The majority of experts distinguish about 30 species in the genus, but only six are widespread in Eurasia [2]. These pests have vast geographic ranges and cause serious damage to forestry in many countries. Examples of harmful *Dendrolimus* species are *D. pini* (Linnaeus [3]), *D. sibiricus* (Tschetverikov [4]) and *D. punctatus* (Walker [5]). They are thought to be the most dangerous and widespread species of this genus [2]. *D. punctatus* is found in the south-eastern part of Eurasia. Outbreaks of this species occur approximately over one million ha each year in China [6]. *D. pini* is widely distributed across Europe, Central Asia, and North Africa [7] and its outbreaks could also cover vast areas. For example, *D. pini* destroyed about 233,000 ha of forest in Poland from 1946 to 1995. Outbreaks of this pest were also registered in Germany and Russia [8].

The Siberian moth, *D. sibiricus*, is one of the most dangerous pests of coniferous forests in Northern Asia. *D. sibiricus* range occupy territories from the coasts of the Okhotsk and the Japanese Seas to the Ural Mountains [9]. The pest was detected in western Russia and it can be identified even in Central Europe [2, 10, 11]. The European and Mediterranean Plant Protection Organization (EPPO) included *D. sibiricus* in its A2 list of pests recommended for quarantine in member countries [12]. Outbreaks of *D. sibiricus* can cause a huge damage to ecosystems over large territories [13]. At least nine outbreaks were reported in the Central Siberia where they covered an area around 10 million hectares during the last century [14].

Two other species, *D. houi* (Lajonquière [15]) and *D. kikuchii* (Matsumura [16]), are most frequently encountered in eastern Asia and are economically important pests of coniferous forests in South China [17]. During outbreak years of *D. houi* from thousands to millions of hectares of forests can be killed [18]. The second species, *D. kikuchii*, is broadly distributed across Southern China and causes serious damage to trees in provinces of this territory. In the Simao Prefecture, the swarming of its adults overlap with the seasonal flight periods of the broadly sympatric species, *D. houi* [19].

Taxonomy and nomenclature of *Dendrolimus* are not entirely established, and there are many species with a controversial taxonomic position. *D. punctatus, D. tabulaeformis* (Tsai & Liu [20]) and *D. spectabilis* (Butler [21]) have an uncertain species status. *D. tabulaeformis* and *D. spectabilis* had been considered as a subspecies of *D. punctatus* [22]. However, all three species were treated as different in other studies [23]. Taxonomic relationship of *D. sibiricus* and *D. superans* (Butler [21]) is also problematic. According to the consensus opinion, *D. sibiricus* and *D. superans* are separate species, although some researchers consider that there is a single species *D. superans* with two subspecies: *Dendrolimus superans sibiricus* Tschetverikov and *Dendrolimus superans albolineatus* Butler [9].

In 2008, a new species, *Dendrolimus kilmez*, was described from Kilmez, Central Russia [2]. The new species was morphologically similar to *D. pini*, and had identical ITS2 nuclear DNA sequences with it, but was very different from *D. pini* according to a 3′ COI mitochondrial DNA marker.

Genetic diversity is an important indicator of the ability of a species or individual populations to adapt to the environment. Species or populations with greater genetic diversity are better adapted to changing environmental conditions [24, 25]. Therefore, they are more likely to expand their habitats and geographic ranges. Studies of genetic variation provide information on the origin and divergence of species and explain their geographical distribution. This information can also help to understand the quarantine measures which should be implemented in cases of pest species [26, 27]. New, genetically homogenous populations can be formed by fast migration to the territories, which are sufficiently distant from the source range [28]. The degree of genetic diversity of the newly formed populations can reach the genetic diversity of source populations as the range expands and population grows [26].

In this paper we studied the genetic structure of populations of eight *Dendrolimus* species widespread in Eurasia. A few original studies exist that address genetic diversity and phylogeny only of individual species or groups of closely related species of *Dendrolimus* [2, 29, 30]. These studies provided information only about populations from western (Europe) and eastern (China) parts of Eurasia; however, a large portion of *D. sibiricus* and *D. pini* ranges lies in Siberia [12, 31]. In addition, in each work different genetic markers were used. This fact complicates the comparison and synthesis of the results of performed researches. The present study used all the information about the genetic diversity of *Dendrolimus* known to date. Specimens from populations of central part of Eurasia were collected for a more complete understanding of the genetic structure of the genus, bringing together geographically separated areas of previous research. We used all the genetic markers previously used in the studies of the genus *Dendrolimus*. That made possible to compare the disparate data from previous studies. We provide the most up-to-date information on the genetic differentiation of populations of the eight the most important *Dendrolimus* species in Eurasia including nearest islands. Species which were investigated in the current work represent the majority of the *Dendrolimus* species across this region. Our study further clarifies taxonomic problems in the genus and gives more complete information on the genetic structure of *D. pini*, *D. sibiricus*, and *D. superans*.

## Methods

### Collection of material, isolation of genomic DNA, PCR amplification and sequencing

Larvae and moths of *D. pini, D. superans*, and *D. sibiricus* were collected in the natural populations across Asian Russia (Siberia, the Russian Far East, and the Sakhalin Island). Localities, number of collected specimens and Genbank accession numbers are listed in Additional file 1. No special permits were required for the described field studies, the localities were not privately owned, and the field studies did not involve endangered or protected species.

Genomic DNA was extracted from insect tissues using the DNeasy Blood & Tissue Kit (QIAGEN, Valencia, CA) in accordance with the manufacturer’s protocol.

Partial mtDNA sequences of COI and COII genes, about 1400 and 600 bp long, correspondingly, and ITS2 sequences (400 bp) were amplified by PCR using specific primer pairs listed in Table 1. For details of the extraction and PCR amplification, PCR purification and sequencing, see Vavilova et al. [32]. The insertion sequences were sequenced on an automated sequencer ABI PrISM 3100 Avant Genetic Analyzer (Applied Biosystems, USA) with a Big Dye terminator sequencing kit (Applied Biosystems, USA) at the SB RAS Genomics Core Facility (Novosibirsk, Russia, http://sequest.niboch.nsc.ru).

**Table 1:** Primers used for PCR amplification of the fragments of nuclear and mitochondrial genes

DNA fragment	Primers	Sequence	Source
3′ end of COI gene	M5	5′- CAACATTTATTTTGATTTTTTGG-3′	[2]
	M3	5′- CCAATGCACTAATCTGCCATATTA-3′	[2]
5′ end of COI gene	911	5′-TTTCTACAAATCATAAAGATATTGG-3′	[44]
	912	5′-TAAACTTCAGGGTGACCAAAAATCA-3′	[44]
Portion of COII gene	C2N	5′- CCACAAATTTCTGAACATTGACCA -3′	[45]
	C2J	5′- AGAGCTTCTCCTTTAATAGAACA -3′	[45]
ITS2	ITS2A	5′- TGTGAACTGCAGGACACAT -3′	[2]
	ITS2B	5′- TATGCTTAAATTGAGGGGGT -3′	[2]

### Database screening

DNA sequences of the COI, COII and ITS2 genes of *Dendrolimus* species were extracted from Genbank database of the National Institutes of Health (NCBI), USA, using “(Dendrolimus[Organism]) AND (COI OR CO1)”, “(Dendrolimus[Organism]) AND (COII OR CO2)” and “(Dendrolimus[Organism]) AND ITS2” as search queries. It should be noted that the European population represented only sequences from Genbank. However, this sequences participated in all comparative analyzes, together with all experimentally obtained samples. Outgroup species for phylogenetic analysis of ITS2 and COII sequences were selected from Genbank using BLASTn (NCBI). Sequences of *Zygaena tamara* and *Zygaena seitzi* were chosen for ITS2. Sequences of *Odontopus sanguinolens* and *Odontopus nigricomis* were chosen for COII. For uniformity to analyses of the mitochondrial markers COI the regions from complete mitochondrial genomes of *Biston panterinaria* and *Phthonandria atrilineata* were used as outgroup [33].

### Sequence analysis

Multiple sequence alignment was performed by MUSCLE algorithm in UGENE 1.12 [34]. Analysis of phylogeny by maximum-likelihood (ML) was carried out in PhyML 3.0 program with default settings and with the aLRT as a topology estimation method [35].

## Results and Discussion

### Database screening, collection of new samples, and sequencing

**Fig. 1:**
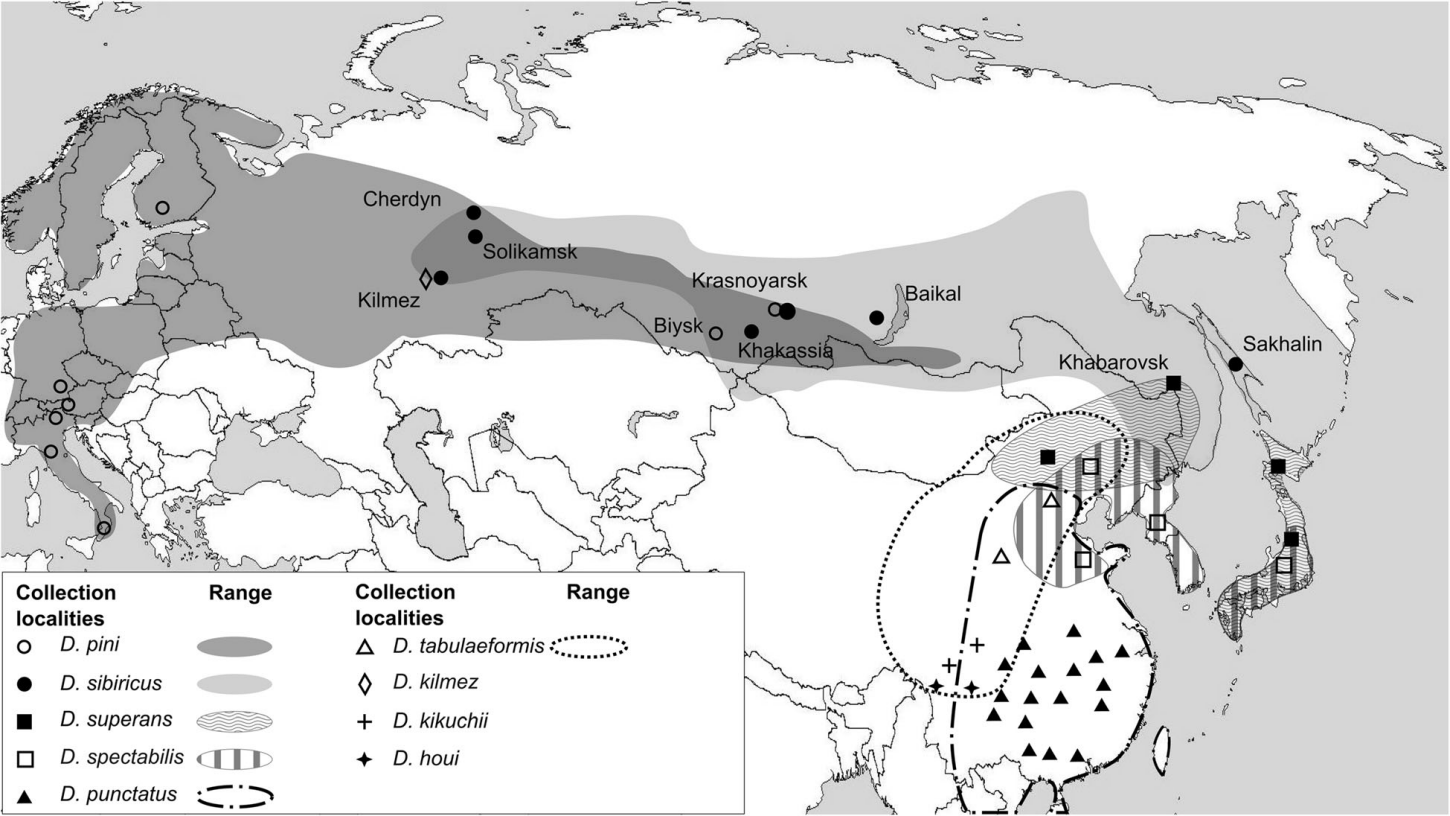
Collection localities and ranges of *Dendrolimus* species analyzed in this work from Europe and Asia. Approximate geographic ranges are indicated by color/texture or circled with a dotted line (http://www.cabi.org/dmpp, CABI 2013) [46]. The overlapping ranges of different species are shown in overlapping textures or shades. Collection localities labeled the settlement name but not the country name signed on the map

In order to assess genetic structure of *Dendrolimus* populations we aimed to retrieve mitochondrial COI and COII sequences, and ITS2 nuclear sequences for eight *Dendrolimus* species currently available in GenBank (NCBI). To integrate disparate data we used seven specimens of *D. pini* from two natural populations of Altai and Krasnoyarsk regions; 17 samples of *D. sibiricus* from three populations of Khakassia, Krasnoyarsk, and Sakhalin regions; and four specimens of *D. superans* from the Far East of Russia (Fig. 1). Total DNA was extracted from each specimen and amplified by PCR with primers specific for COI (5′ and 3′ ends) and COII mitochondrial genes, and for ITS2 nuclear ribosomal DNA gene. Totally, 112 DNA fragments were isolated and sequenced.

The source and localities for all obtained sequences are shown in Table 2 and the total number of COI, COII, and ITS sequences used in the present work are summarized in Table 3. The total length of the DNA fragments was 590, 728, 538 and 504 bp for 5′ COI, 3′ COI, COII, and ITS2, correspondingly.

**Table 2:** Collection localities, sources, and the sequence types of *Dendrolimus* species included in this study

Species	Locality	Sequence type	Source
*D. pini*	Finland	COI 3′, ITS2	[2]
	Germany	COI 5′	GU654860, JF415336-JF415341
	Italy	COI 5′	GU688542, HM914063, JF860056
	Biysk, Russia	COI 5′, COI 3′, ITS2, COII	the present work
	Krasnoyarsk, Russia	COI 5′, COI 3′, ITS2, COII	the present work
	Kilmez, Russia	COI 3′, ITS 2	[2]
*D. sibiricus*	Cherdyn, Russia	COI 3′, ITS 2	[2]
	Kilmez, Russia	COI 3′, ITS 2	[2]
	Baikal, Russia	COI 3′, ITS 2	[2]
	Solikamsk, Russia	COI 3′, ITS 2	[2]
	Krasnoyarsk, Russia	COI 5′, COI 3′, ITS2, COII	the present work
	Khakassia, Russia	COI 5′, COI 3′, ITS2, COII	the present work
	Sakhalin, Russia	COI 5′, COI 3′, ITS2, COII	the present work
*D. superans*	Japan	COI 3′, ITS 2	[2]
	China	COI 5′, ITS 2	[29, 30]
	Khabarovsk, Russia	COI 5′, COI 3′, ITS2, COII	the present work
*D. spectabilis*	Japan	ITS 2	[2]
	China	COI 5′, COI 3′, ITS2, COII	[29, 30, 33]
	South Korea	COI 5′	KC135936; JN087390
*D. punctatus*	China	COI 5′, COI 3′, ITS2, COII	[29, 30, 33]
*D. tabulaeformis*	China	COI 5′, COI 3′, ITS2, COII	[29, 30, 26]
*D. houi*	China	COI 5′, COI 3′, ITS 2, COII	[29, 30]
*D. kikuchii*	China	COI 5′, ITS 2, COII	[29, 30]

**Table 3:** Number and types of *Dendrolimus* sequences analyzed in this work

Species	5′ COI	3′ COI	ITS2	COII	Total
*D. pini*	17	15	15	7	54
*D. sibiricus*	17	28	30	17	92
*D. superans*	18	14	23	4	59
*D. spectabilis*	25	2	14	2	43
*D. punctatus*	91	4	25	4	124
*D. tabulaeformis*	43	2	27	2	74
*D. kikuchii*	32	0	16	4	52
*D. houi*	14	2	10	4	30
					528

### Phylogeny of the Dendrolimus species

**Fig. 2:**
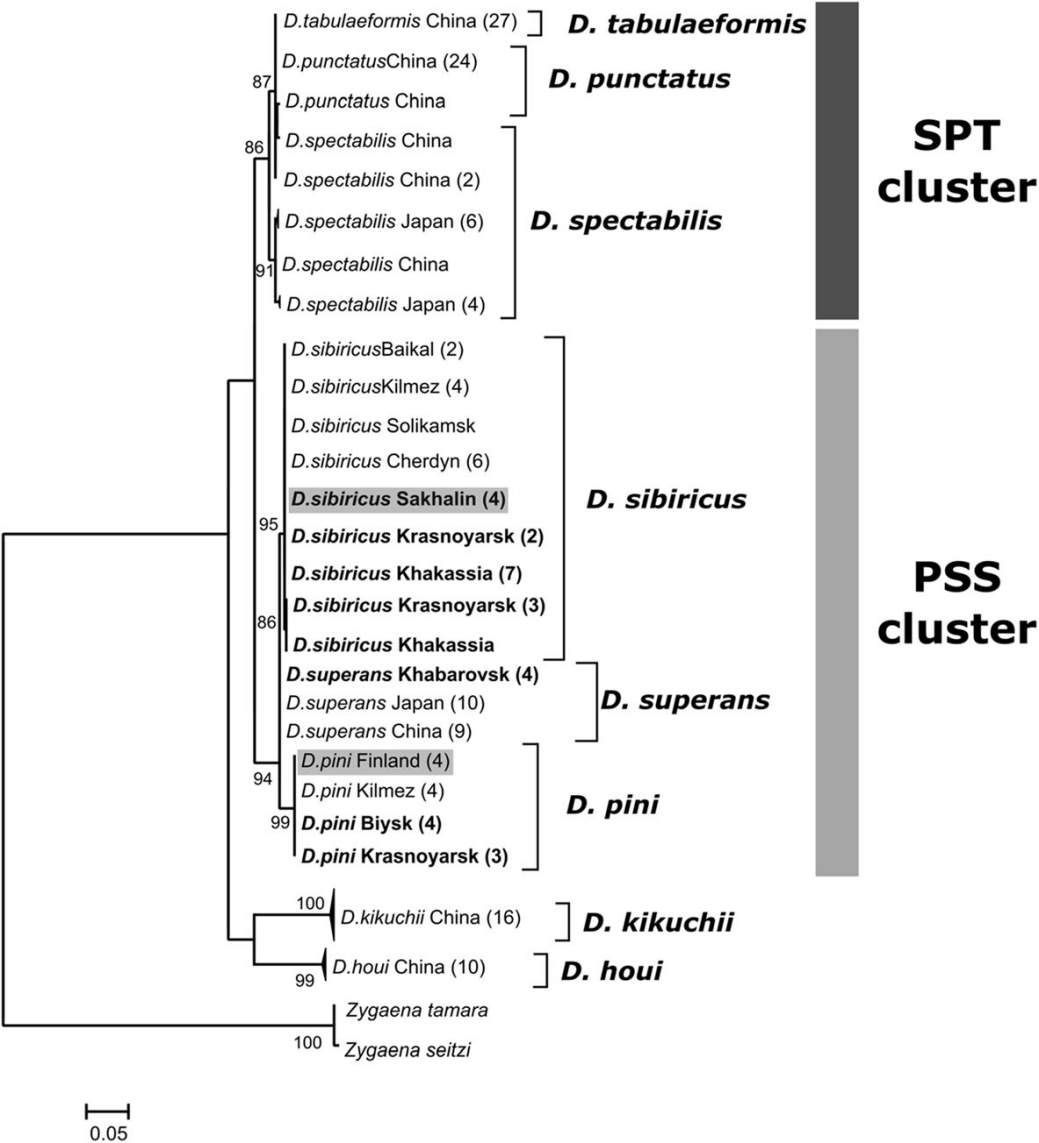
Phylogeny of *Dendrolimus* based on ITS2 nuclear sequences. Names of the samples that were obtained in the present study are indicated in bold. SPT, cluster of closely related species: *D. spectabilis*, *D. punctatus* and *D. tabulaeformis*. PSS, cluster of closely related species: *D. pini*, *D. sibiricus* and *D. superans.* Specimens whose position was in disagreement with the accepted taxonomy are shown in gray. Sequences of the *Zygaena* genus were used as the outgroup. The coefficients near the tree nodes represent the statistical support for respective branches. The coefficients below 80 are not shown in the picture

**Fig. 3:**
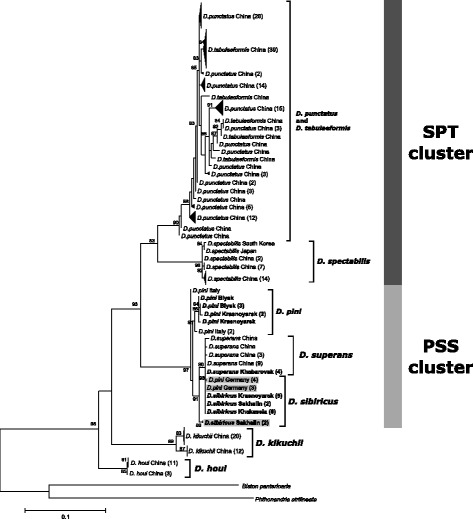
Phylogeny of *Dendrolimus* based on 5′-end portion of COI gene. Specimens obtained in the present study are shown in bold. SPT, cluster of closely related species: *D. spectabilis*, *D. punctatus* and *D. tabulaeformis*. PSS, cluster of closely related species: *D. pini*, *D. sibiricus* and *D. superans.* Specimens whose position was in disagreement with the accepted taxonomy are shown in gray. Sequences of the *Biston* and *Phthonandria* genus were used as the outgroup. The coefficients near the tree nodes represent the statistical support for respective branches. The coefficients below 80 are not shown in the picture

**Fig. 4:**
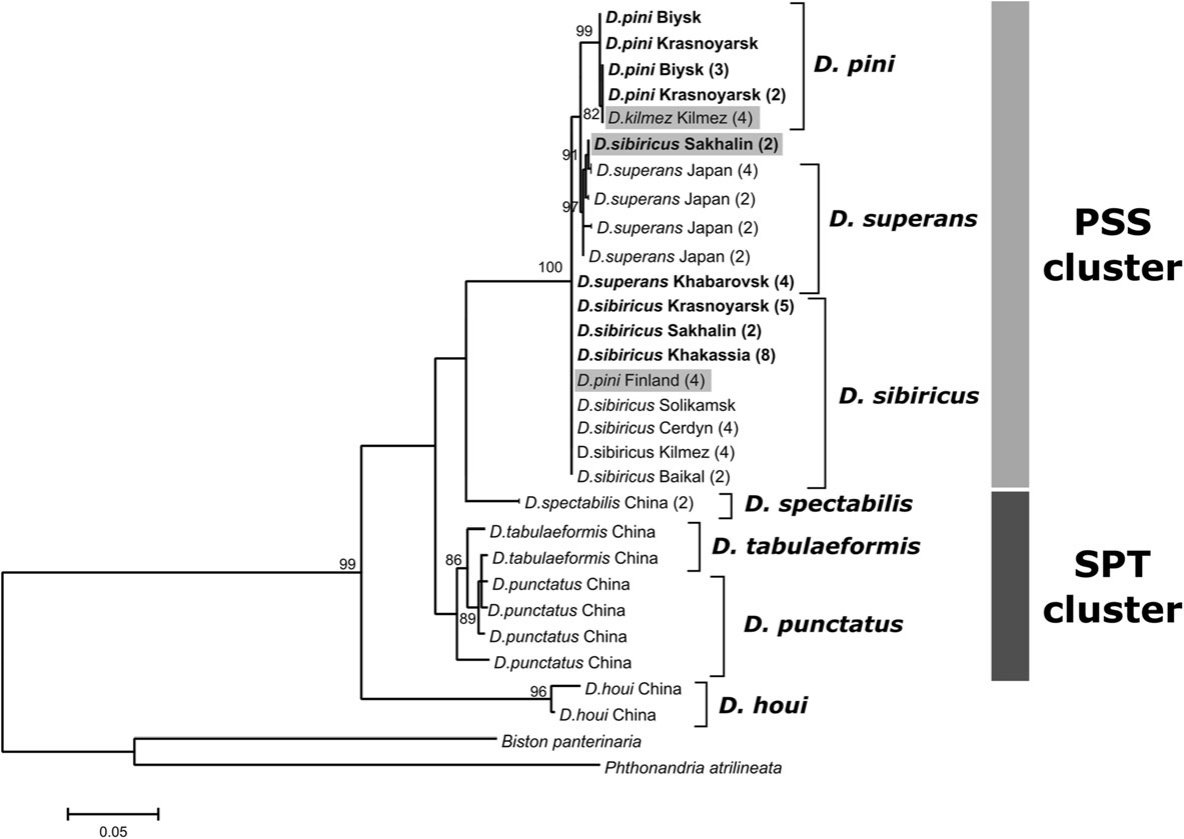
Phylogeny of *Dendrolimus* based on 3′-end portion of COI gene. Specimens obtained in the present study are shown in bold. SPT, cluster of closely related species: *D. spectabilis*, *D. punctatus* and *D. tabulaeformis*. PSS, cluster of closely related species: *D. pini*, *D. sibiricus* and *D. superans.* Specimens whose position was in disagreement with the accepted taxonomy are shown in gray. Sequences of the *Biston* and *Phthonandria* genus were used as the outgroup. The coefficients near the tree nodes represent the statistical support for respective branches. The coefficients below 80 are not shown in the picture

**Fig. 5:**
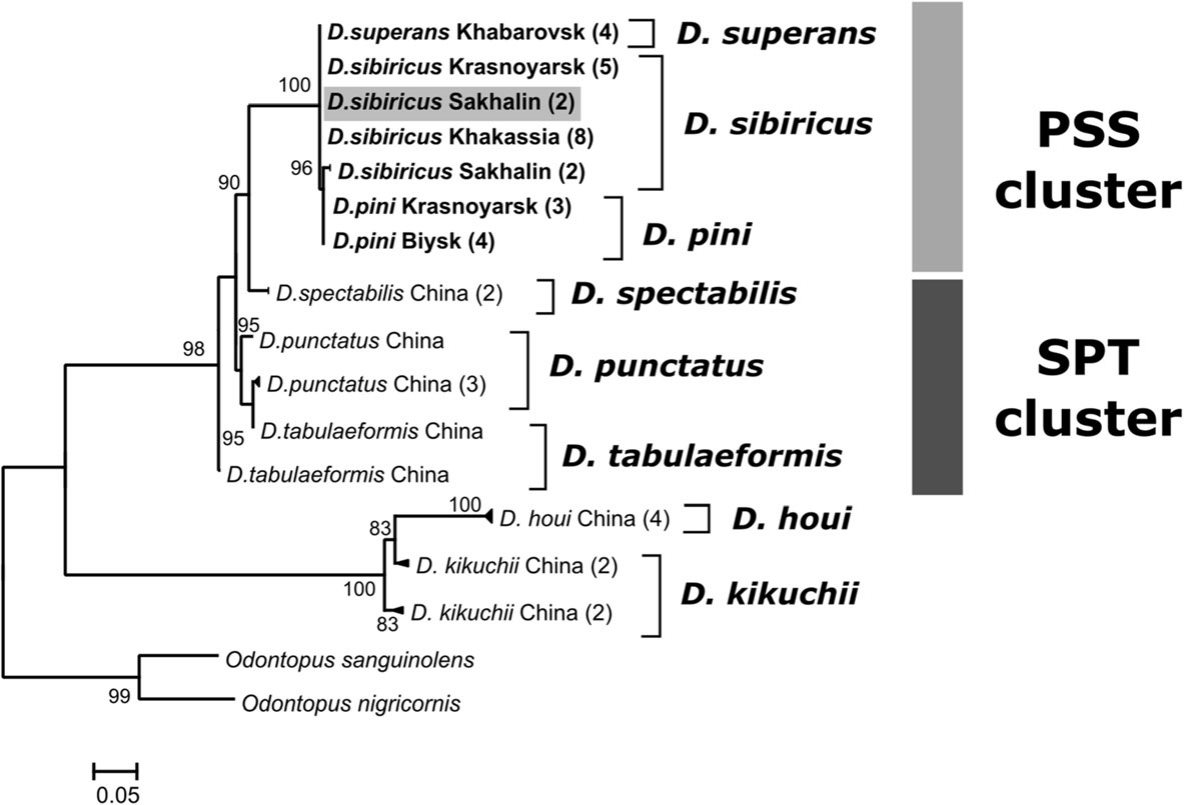
Phylogeny of *Dendrolimus* based on a portion of COII gene. Specimens obtained in the present study are shown in bold. SPT, cluster of closely related species: *D. spectabilis*, *D. punctatus* and *D. tabulaeformis*. PSS, cluster of closely related species: *D. pini*, *D. sibiricus* and *D. superans.* Specimens whose position was in disagreement with the accepted taxonomy are shown in gray. Sequences of the *Odontopus* genus were used as the outgroup. The coefficients near the tree nodes represent the statistical support for respective branches. The coefficients below 80 are not shown in the picture

ML phylogenetic trees were reconstructed based on multiple sequence alignments of four different DNA sequences: ITS2, 5′ COI, 3′ COI and COII of *Dendrolimus* species (Figs. 2, 3, 4 and 5). Two clusters of closely related species were distinguished on the all phylogenetic trees. The first cluster (SPT, by the first letter in species’ name) is formed by *D. spectabilis*, *D. punctatus,* and *D. tabulaeformis. D. spectabilis* is a quite clearly separated species in the SPT cluster in contrast to *D. punctatus* and *D. tabulaeformis* which form a closely related group. The second cluster (PSS, by the first letter in species’ name) is formed by three species: *D. pini*, *D. sibiricus,* and *D. superans. D. superans* is more closely related to *D. sibiricus* than to *D. pini*. Two species, *D. kikuchii* and *D. houi,* are the most distant from all other *Dendrolimus* species, and the most basal species in the genus.

### Phylogenetic relationships and phylogeography of species in the SPT cluster

*D. spectabilis*, *D. tabulaeformis* and *D. punctatus* species can be isolated in the separate cluster based on the all analyzed markers. According to mitochondrial sequences *D. spectabilis* are clearly allocated into the group which is close to *D. tabulaeformis* and *D. punctatus* but which evolved independently of them (Figs. 3, 4 and 5). In contrast, *D. tabulaeformis* is not distinguishable from the *D. punctatus* on the all COI and ITS2 phylogenetic trees supporting its subspecies status [33]. In concordance with the ITS2 tree, SPT cluster looks more homogeneous due to the lower resolution of nuclear markers (Fig. 2). A similar pattern of relationships between species of the SPT cluster has been shown by a series of other molecular barcoding methods [36]. Furthermore, recent studies of mitochondrial genomes of these species provide an analogous results [33].

Species of the SPT cluster have highly overlapping geographic ranges (Fig. 1). Therefore, this situation can be explained by absence of geographical isolation among these species, which could lead to the constant gene flow even between relatively distant populations of different species; consequently, a clearly separated, independent population could not be formed. A high degree of genetic variability is a common feature of populations with a continuous range (absence of geographical isolation), high abundance, and an ability to disperse over large distances (typical for flying insects). All this allows for gene flow between even relatively distant groups that results in genetic heterogeneity of individual populations [26, 37, 38]. This situation is applicable to populations of *D. tabulaeformis*, and *D. punctatus*, where a high degree of genetic variability is confirmed by phylogenetic analysis of mitochondrial and nuclear DNA sequences (Figs. 2, 3, 4 and 5).

### Phylogenetic relationships and phylogeography of species in the PSS cluster

#### *D. sibiricus* and *D. superans*

According to the recent review [2], *D. sibiricus* and *D. superans* are currently considered separate species. The same was declared by EPPO at 2005, based on an unidentified international opinion [12]. However, other reviews considered *D. sibiricus* to be a subspecies of *D. superans* [9, 39].

Our results demonstrate that *D. sibiricus* and *D. superans* are clearly distinguished from each other based on the phylogenetic analysis of ITS2 sequences (Fig. 2). This situation confirms the first viewpoint on *D. sibiricus* as on the separate species. At the same time, continental populations of these two species are identical according to the mitochondrial markers (Figs. 3, 4 and 5). This result supports *D. sibiricus* as a subspecies. Furthermore, sequences of both species from island populations formed a separate clearly distinguishable group according with the 3′ COI tree (Fig. 4). Continental populations of *D. superans* are more divergent from the Japanese populations of the same species than from continental populations of *D. sibiricus*. Consequently, divergence between continental and island populations within a species exceeds interspecies divergence.

It is clear that populations of *D. sibiricus* and *D. superans* shared the nearest common ancestor with respect to other PSS species, as evidenced by ITS2 and COI phylogenies (Figs. 2, 3 and 4). Considering that current range of *D. sibiricus* exceeds the *D. superans* range and the *D. sibiricus* populations are now dispersed in Northeastern Asia, it can be suggested that isolation of *D. sibiricus* as a distinct, but closely related to *D. superans* species, is a matter of short time. Rozhkov [40] also considered *D. sibiricus* to be a relatively young and “progressive” species with unstable population dynamics.

Both nuclear and mitochondrial sequences of *D. sibiricus* populations are completely identical throughout the species range, except of the samples from Sakhalin Island. Such homogeneity can be explained by periodic pandemic outbreaks of *D. sibiricus,* which can cover up to 10 million hectares with intensive migrations throughout outbreak area and neighboring territories [14]. Based on the 5′ COI, 3′ COI and COII sequence analyses, there are at least two mitochondrial haplotypes of *D. sibiricus* in the Sakhalin Island. One of these haplotypes is identical to the continental variant, while another one is unique to Sakhalin.

Similar situation was observed for *D. superans*. Japanese populations of this species are represented by four unique mitochondrial haplotypes based on 3′ COI tree (Fig. 4). Two of these haplotypes are similar to those of the continental populations of *D. superans*, and two others are similar to the unique *D. sibiricus* haplotype from Sakhalin.

Effect of geographic isolation on genetic diversity within populations of one or more closely related species has been shown repeatedly in various studies on both interspecific and intraspecific variability in insects [37, 41, 42]. A general pattern is seen in all described cases: gene flow is common between closely related species of insects with overlapping ranges or common plant hosts. Frequent gene flow results in the formation of genetically homogeneous populations of different morphotypes at the junction of the common ranges and habitats and, conversely, in an increase in interspecific divergence when moving away from them, like in SPT cluster. Isolated populations sever all contacts with source populations, and start forming genetically isolated groups, with at least one haplotype represented in the presence of a more rigorous barrier than isolation by distance (such as mountains or watersheds), but they are still morphologically identical to the source species [37]. A similar situation is observed in the case of the continental and island populations of *D. superans* and *D. sibiricus.*

#### *D. pini, D. sibiricus*, and *D. kilmez*

*D. pini* sequences formed the isolated branch in PSS cluster on the all analyzed trees. All studied *D. pini* from the populations located at large distances from each other showed no genetic variability in ITS2 (Fig. 2). There are differences in mitochondrial gene sequences both between distant populations and within the same population of *D. pini* (Fig. 3). *D. pini* populations from Finland were separated from all other populations of this species on the 3 ′COI phylogenetic tree. They grouped together with the samples of *D. sibiricus* from mainland populations (Fig. 4). Thus, *D. pini* from Finland are identical to *D. sibiricus* according to mitochondrial markers, but identical to the other *D. pini* specimens according to nuclear markers (Figs. 2 and 4). In addition, *D. pini* from Europe (Germany, Bavaria) were also identical to *D. sibiricus* according to the 5′ COI phylogeny (Fig. 3). Therefore, some European populations contain *D. pini* individuals with the mitochondrial marker sequences completely identical to *D. sibiricus*.

In 2008, a phylogenetic study of *Dendrolimus* was conducted [2]. In this work, populations of different *Dendrolimus* species from Japan, Russia (Kilmez and Solikamsk) and Finland were investigated. The four samples from Finland were identified as *D. pini* and the four samples from Kilmez (Russia), as a new species named *D. kilmez*. The new species was very similar to *D. pini* morphologically, but differed genetically from the Finnish populations of *D. pini*, based on the 3′ COI sequence analysis [2]. Our phylogenetic analysis, in addition to the Finnish populations of *D. pini*, included specimens from Italy, Germany and Russia (Siberia). *D. kilmez* clustered together with *D. pini* specimens, which were added in the current work (Figs. 2 and 4). Therefore, according to both nuclear and mitochondrial markers, the *D. kilmez* individuals are members of the *D. pini* species. In contrast, specimens of *D. pini* from Finland clustered together with *D. sibiricus* on the 3′ COI phylogenetic tree (Fig. 4), but not with the other *D. pini*. However, according to the ITS2 phylogeny, *D. pini* from Finland clearly belong to *D. pini* (Fig. 2). This means that mitochondrial differences of the Finnish individuals differ from other *D. pini* caused the incorrect assignment of Kilmez individuals to a separate species. Eventually specimens named *D. kilmez* represents the typical *D. pini* and Finnish *D. pini* specimens differ from other *D. pini* on the mitochondrial level.

The presence of mitochondrial DNA sequences of *D. sibiricus* simultaneously with the nuclear DNA sequences of *D. pini* in the Finnish and Germany individuals can be explained by the possibility of cross-hybridization between *D. sibiricus* and *D. pini. D. sibiricus* and *D. pini* have overlapping ranges through nearly all south of Western and Eastern Siberia (Fig. 1). Their breeding seasons also overlap and their sex pheromones are very close. *D. sibiricus* can occure in the same habitats with *D. pini* (e.g., as it takes place in the *Pinus sylvestris* forests in the foothills of Eastern Sayan Mountains) [13]. During the outbreaks, both species could expand their ranges manyfold [31]. Thus, formation of *D. sibiricus* – *D. pini* hybrids in nature seems not so improbable.

## Conclusion

In the present work we investigated genetic diversity of eight *Dendrolimus* species in Eurasia by integration of our own data with all that obtained on different genetic markers from different studies. While only supporting some of the appreciated ideas on evolutionary relationships among *D. spectabilis*, *D. punctatus* and *D. tabulaeformis* (SPT cluster), we unraveled the status of *D. kilmez* as a new species and showed cases of integration of *D. sibiricus* mitochondrial DNA sequences to European populations of its close relative *D. pini*. Subspecies status of *D. tabulaeformis* was supported. Almost uniform genetic variability of continental *D. sibiricus* populations was shown, suggesting its impetuous spread to the west of Eurasia. Thereby, our data support appreciation of *D. sibiricus* as an important pest for Europe and its inclusion into A2 list of pests recommended for regulation as quarantine pests [43].

## Additional file

Additional file 1: Localities, number of collected *Dendrolimus* specimens and their Genbank accession numbers. (PDF 257 kb)
