# Supplementary material for: Genetic diversity among eight Dendrolimus species in Eurasia (Lepidoptera: Lasiocampidae) inferred from mitochondrial COI and COII, and nuclear ITS2 markers
Source: BMC Genet. 2016 Dec 22;17(Suppl 3):157. doi: 10.1186/s12863-016-0463-5 (PMC5249024; doi:10.1186/s12863-016-0463-5)
Supplement: Additional file 1: — Localities, number of collected Dendrolimus specimens and their Genbank accession numbers. (PDF 257 kb) [file 12863_2016_463_MOESM1_ESM.pdf]

Genetic diversity among eight *Dendrolimus* species in Eurasia (Lepidoptera: Lasiocampidae) inferred from mitochondrial COI and COII, and nuclear ITS2 markers

BMC Genetics

Alexandr Kononov, Kirill Ustyantsev, Baode Wang, Victor C. Mastro, Victor Fet, Alexander Blinov, Yuri Baranchikov
Corresponding author: Alexander Kononov, e-mail: kononov.nov@mail.ru
Institute of Cytology and Genetics, the Siberian Branch of the Russian Academy of Science. 10 Prospekt Lavrentyeva, Novosibirsk 630090, Russia

Species	Locality	Sequence Type	Source	isolate (present work)	Accession number
D. pini	Finland, Tammisaari	COI 3’	Mikkola & Ståhls, 2008		AM946710
D. pini	Finland, Tammisaari	COI 3’	Mikkola & Ståhls, 2008		AM946708
D. pini	Finland, Tammisaari	COI 3’	Mikkola & Ståhls, 2008		AM946709
D. pini	Finland, Tammisaari	COI 3’	Mikkola & Ståhls, 2008		AM946711
D. pini	Finland, Tammisaari	ITS2	Mikkola & Ståhls, 2008		AM946723
D. pini	Finland, Tammisaari	ITS2	Mikkola & Ståhls, 2008		AM946724
D. pini	Finland, Tammisaari	ITS2	Mikkola & Ståhls, 2008		AM946725
D. pini	Finland, Tammisaari	ITS2	Mikkola & Ståhls, 2008		AM946726
D. pini	Germany, Bavaria	COI 5’	Hausmann, 2011		GU654860
D. pini	Germany, Bavaria	COI 5’	Hausmann et al., 2012		JF415336
D. pini	Germany, Bavaria	COI 5’	Hausmann et al., 2013		JF415337
D. pini	Germany, Bavaria	COI 5’	Hausmann et al., 2014		JF415338
D. pini	Germany, Bavaria	COI 5’	Hausmann et al., 2015		JF415339
D. pini	Germany, Bavaria	COI 5’	Hausmann et al., 2016		JF415340
D. pini	Germany, Bavaria	COI 5’	Hausmann et al., 2017		JF415341
D. pini	Italy	COI 5’	Hausmann, 2011		GU688542
D. pini	Italy	COI 5’	Hausmann, 2010		HM914063
D. pini	Italy	COI 5’	Huemer, 2010		JF860056
D. pini	Russia, Altai, Biysk area	COI 5’	the present work	DP41	KJ007764
D. pini	Russia, Altai, Biysk area	COI 5’	the present work	DP42	KJ007765
D. pini	Russia, Altai, Biysk area	COI 5’	the present work	DP43	KJ007766
D. pini	Russia, Altai, Biysk area	COI 5’	the present work	DP44	KJ007767
D. pini	Russia, Altai, Biysk area	COI 3’	the present work	DP41	KJ007764
D. pini	Russia, Altai, Biysk area	COI 3’	the present work	DP42	KJ007765
D. pini	Russia, Altai, Biysk area	COI 3’	the present work	DP43	KJ007766
D. pini	Russia, Altai, Biysk area	COI 3’	the present work	DP44	KJ007767
D. pini	Russia, Altai, Biysk area	ITS2	the present work	DP41	KJ007760
D. pini	Russia, Altai, Biysk area	ITS2	the present work	DP42	KJ007761
D. pini	Russia, Altai, Biysk area	ITS2	the present work	DP43	KJ007762
D. pini	Russia, Altai, Biysk area	ITS2	the present work	DP44	KJ007763
D. pini	Russia, Altai, Biysk area	COII	the present work	DP41	KJ007796
D. pini	Russia, Altai, Biysk area	COII	the present work	DP42	KJ007797
D. pini	Russia, Altai, Biysk area	COII	the present work	DP43	KJ007798
D. pini	Russia, Altai, Biysk area	COII	the present work	DP44	KJ007799
D. pini	Russia, Krasnoyarsk, Anashsky bor	COI 5’	the present work	DP61	KJ007768
D. pini	Russia, Krasnoyarsk, Anashsky bor	COI 5’	the present work	DP62	KJ007769
D. pini	Russia, Krasnoyarsk, Anashsky bor	COI 5’	the present work	DP64	KJ007770
D. pini	Russia, Krasnoyarsk, Anashsky bor	COI 3’	the present work	DP61	KJ007768
D. pini	Russia, Krasnoyarsk, Anashsky bor	COI 3’	the present work	DP62	KJ007769
D. pini	Russia, Krasnoyarsk, Anashsky bor	COI 3’	the present work	DP64	KJ007770
D. pini	Russia, Krasnoyarsk, Anashsky bor	ITS2	the present work	DP61	KJ007759
D. pini	Russia, Krasnoyarsk, Anashsky bor	ITS2	the present work	DP62	KJ007758
D. pini	Russia, Krasnoyarsk, Anashsky bor	ITS2	the present work	DP64	KJ007757
D. pini	Russia, Krasnoyarsk, Anashsky bor	COII	the present work	DP61	KJ007808
D. pini	Russia, Krasnoyarsk, Anashsky bor	COII	the present work	DP62	KJ007809
D. pini	Russia, Krasnoyarsk, Anashsky bor	COII	the present work	DP64	KJ007810
D. pini	Russia, Kilmezh	COI 3’	Mikkola & Ståhls, 2008		AM946693
D. pini	Russia, Kilmezh	COI 3’	Mikkola & Ståhls, 2008		AM946705
D. pini	Russia, Kilmezh	COI 3’	Mikkola & Ståhls, 2008		AM946706
D. pini	Russia, Kilmezh	COI 3’	Mikkola & Ståhls, 2008		AM946707
D. pini	Russia, Kilmezh	ITS 2	Mikkola & Ståhls, 2008		AM946727
D. pini	Russia, Kilmezh	ITS 2	Mikkola & Ståhls, 2008		AM946728
D. pini	Russia, Kilmezh	ITS 2	Mikkola & Ståhls, 2008		AM946729
D. pini	Russia, Kilmezh	ITS 2	Mikkola & Ståhls, 2008		AM946730
D. sibiricus	Russia, Cherdyn	COI 3’	Mikkola & Ståhls, 2008		AM946694
D. sibiricus	Russia, Cherdyn	COI 3’	Mikkola & Ståhls, 2008		AM946695
D. sibiricus	Russia, Cherdyn	COI 3’	Mikkola & Ståhls, 2008		AM946699
D. sibiricus	Russia, Cherdyn	COI 3’	Mikkola & Ståhls, 2008		AM946700
D. sibiricus	Russia, Cherdyn	ITS 2	Mikkola & Ståhls, 2008		AM946744
D. sibiricus	Russia, Cherdyn	ITS 2	Mikkola & Ståhls, 2008		AM946745

D. sibiricus	Russia, Cherdyn	ITS 2	Mikkola & Ståhls, 2008		AM946747
D. sibiricus	Russia, Cherdyn	ITS 2	Mikkola & Ståhls, 2008		AM946748
D. sibiricus	Russia, Cherdyn	ITS 2	Mikkola & Ståhls, 2008		AM946749
D. sibiricus	Russia, Cherdyn	ITS 2	Mikkola & Ståhls, 2008		AM946750
D. sibiricus	Russia, Kilmezh	COI 3’	Mikkola & Ståhls, 2008		AM946697
D. sibiricus	Russia, Kilmezh	COI 3’	Mikkola & Ståhls, 2008		AM946698
D. sibiricus	Russia, Kilmezh	COI 3’	Mikkola & Ståhls, 2008		AM946701
D. sibiricus	Russia, Kilmezh	COI 3’	Mikkola & Ståhls, 2008		AM946702
D. sibiricus	Russia, Kilmezh	ITS 2	Mikkola & Ståhls, 2008		AM946751
D. sibiricus	Russia, Kilmezh	ITS 2	Mikkola & Ståhls, 2008		AM946752
D. sibiricus	Russia, Kilmezh	ITS 2	Mikkola & Ståhls, 2008		AM946753
D. sibiricus	Russia, Kilmezh	ITS 2	Mikkola & Ståhls, 2008		AM946756
D. sibiricus	Russia, Baikal	COI 3’	Mikkola & Ståhls, 2008		AM946703
D. sibiricus	Russia, Baikal	COI 3’	Mikkola & Ståhls, 2008		AM946704
D. sibiricus	Russia, Baikal	ITS 2	Mikkola & Ståhls, 2008		AM946754
D. sibiricus	Russia, Baikal	ITS 2	Mikkola & Ståhls, 2008		AM946755
D. sibiricus	Russia, Solikamsk	COI 3’	Mikkola & Ståhls, 2008		AM946696
D. sibiricus	Russia, Solikamsk	ITS 2	Mikkola & Ståhls, 2008		AM946746
D. sibiricus	Russia, Krasnoyarsk, Anashsky bor	COI 5’	the present work	DS51	KJ007773
D. sibiricus	Russia, Krasnoyarsk, Anashsky bor	COI 5’	the present work	DS52	KJ007774
D. sibiricus	Russia, Krasnoyarsk, Anashsky bor	COI 5’	the present work	DS53	KJ007775
D. sibiricus	Russia, Krasnoyarsk, Anashsky bor	COI 5’	the present work	DS54	KJ007776
D. sibiricus	Russia, Krasnoyarsk, Anashsky bor	COI 5’	the present work	DP63	KJ007777
D. sibiricus	Russia, Krasnoyarsk, Anashsky bor	COI 3’	the present work	DS51	KJ007773
D. sibiricus	Russia, Krasnoyarsk, Anashsky bor	COI 3’	the present work	DS52	KJ007774
D. sibiricus	Russia, Krasnoyarsk, Anashsky bor	COI 3’	the present work	DS53	KJ007775
D. sibiricus	Russia, Krasnoyarsk, Anashsky bor	COI 3’	the present work	DS54	KJ007776
D. sibiricus	Russia, Krasnoyarsk, Anashsky bor	COI 3’	the present work	DP63	KJ007777
D. sibiricus	Russia, Krasnoyarsk, Anashsky bor	ITS 2	the present work	DS51	KJ007740
D. sibiricus	Russia, Krasnoyarsk, Anashsky bor	ITS 2	the present work	DS52	KJ007741
D. sibiricus	Russia, Krasnoyarsk, Anashsky bor	ITS 2	the present work	DS53	KJ007743
D. sibiricus	Russia, Krasnoyarsk, Anashsky bor	ITS 2	the present work	DS54	KJ007742
D. sibiricus	Russia, Krasnoyarsk, Anashsky bor	ITS 2	the present work	DP63	KJ007745
D. sibiricus	Russia, Krasnoyarsk, Anashsky bor	COII	the present work	DS51	KJ007800
D. sibiricus	Russia, Krasnoyarsk, Anashsky bor	COII	the present work	DS52	KJ007801
D. sibiricus	Russia, Krasnoyarsk, Anashsky bor	COII	the present work	DS53	KJ007802
D. sibiricus	Russia, Krasnoyarsk, Anashsky bor	COII	the present work	DS54	KJ007803
D. sibiricus	Russia, Krasnoyarsk, Anashsky bor	COII	the present work	DP63	KJ007810
D. sibiricus	Russia, Khakassia, Chernoe lake	COI 5’	the present work	DS31	KJ007782
D. sibiricus	Russia, Khakassia, Chernoe lake	COI 5’	the present work	DS32	KJ007783
D. sibiricus	Russia, Khakassia, Chernoe lake	COI 5’	the present work	DS33	KJ007784
D. sibiricus	Russia, Khakassia, Chernoe lake	COI 5’	the present work	DS34	KJ007785
D. sibiricus	Russia, Khakassia, Chernoe lake	COI 5’	the present work	DS81	KJ007788
D. sibiricus	Russia, Khakassia, Chernoe lake	COI 5’	the present work	DS82	KJ007789
D. sibiricus	Russia, Khakassia, Chernoe lake	COI 5’	the present work	DS83	KJ007790
D. sibiricus	Russia, Khakassia, Chernoe lake	COI 5’	the present work	DS84	KJ007791
D. sibiricus	Russia, Khakassia, Chernoe lake	COI 3’	the present work	DS31	KJ007782
D. sibiricus	Russia, Khakassia, Chernoe lake	COI 3’	the present work	DS32	KJ007783
D. sibiricus	Russia, Khakassia, Chernoe lake	COI 3’	the present work	DS33	KJ007784
D. sibiricus	Russia, Khakassia, Chernoe lake	COI 3’	the present work	DS34	KJ007785
D. sibiricus	Russia, Khakassia, Chernoe lake	COI 3’	the present work	DS81	KJ007788
D. sibiricus	Russia, Khakassia, Chernoe lake	COI 3’	the present work	DS82	KJ007789
D. sibiricus	Russia, Khakassia, Chernoe lake	COI 3’	the present work	DS83	KJ007790
D. sibiricus	Russia, Khakassia, Chernoe lake	COI 3’	the present work	DS84	KJ007791
D. sibiricus	Russia, Khakassia, Chernoe lake	ITS 2	the present work	DS31	KJ007736
D. sibiricus	Russia, Khakassia, Chernoe lake	ITS 2	the present work	DS32	KJ007737
D. sibiricus	Russia, Khakassia, Chernoe lake	ITS 2	the present work	DS33	KJ007738
D. sibiricus	Russia, Khakassia, Chernoe lake	ITS 2	the present work	DS34	KJ007739
D. sibiricus	Russia, Khakassia, Chernoe lake	ITS 2	the present work	DS81	KJ007749
D. sibiricus	Russia, Khakassia, Chernoe lake	ITS 2	the present work	DS82	KJ007750
D. sibiricus	Russia, Khakassia, Chernoe lake	ITS 2	the present work	DS83	KJ007751
D. sibiricus	Russia, Khakassia, Chernoe lake	ITS 2	the present work	DS84	KJ007752
D. sibiricus	Russia, Khakassia, Chernoe lake	COII	the present work	DS31	KJ007792
D. sibiricus	Russia, Khakassia, Chernoe lake	COII	the present work	DS32	KJ007793
D. sibiricus	Russia, Khakassia, Chernoe lake	COII	the present work	DS33	KJ007794
D. sibiricus	Russia, Khakassia, Chernoe lake	COII	the present work	DS34	KJ007795
D. sibiricus	Russia, Khakassia, Chernoe lake	COII	the present work	DS81	KJ007816
D. sibiricus	Russia, Khakassia, Chernoe lake	COII	the present work	DS82	KJ007817
D. sibiricus	Russia, Khakassia, Chernoe lake	COII	the present work	DS83	KJ007818
D. sibiricus	Russia, Khakassia, Chernoe lake	COII	the present work	DS84	KJ007819
D. sibiricus	Russia, Sakhalin, Krasnogorsk area	COI 5’	the present work	DS71	KJ007771

D. sibiricus	Russia, Sakhalin, Krasnogorsk area	COI 5’	the present work	DS72	KJ007772
D. sibiricus	Russia, Sakhalin, Krasnogorsk area	COI 5’	the present work	DS73	KJ007786
D. sibiricus	Russia, Sakhalin, Krasnogorsk area	COI 5’	the present work	DS74	KJ007787
D. sibiricus	Russia, Sakhalin, Krasnogorsk area	COI 3’	the present work	DS71	KJ007771
D. sibiricus	Russia, Sakhalin, Krasnogorsk area	COI 3’	the present work	DS72	KJ007772
D. sibiricus	Russia, Sakhalin, Krasnogorsk area	COI 3’	the present work	DS73	KJ007786
D. sibiricus	Russia, Sakhalin, Krasnogorsk area	COI 3’	the present work	DS74	KJ007787
D. sibiricus	Russia, Sakhalin, Krasnogorsk area	ITS2	the present work	DS71	KJ007744
D. sibiricus	Russia, Sakhalin, Krasnogorsk area	ITS2	the present work	DS72	KJ007746
D. sibiricus	Russia, Sakhalin, Krasnogorsk area	ITS2	the present work	DS73	KJ007747
D. sibiricus	Russia, Sakhalin, Krasnogorsk area	ITS2	the present work	DS74	KJ007748
D. sibiricus	Russia, Sakhalin, Krasnogorsk area	COII	the present work	DS71	KJ007815
D. sibiricus	Russia, Sakhalin, Krasnogorsk area	COII	the present work	DS72	KJ007813
D. sibiricus	Russia, Sakhalin, Krasnogorsk area	COII	the present work	DS73	KJ007814
D. sibiricus	Russia, Sakhalin, Krasnogorsk area	COII	the present work	DS74	KJ007812
D. superans	Japan, Nagano, Tazawa	COI 3’	Mikkola & Ståhls, 2008		AM946712
D. superans	Japan, Nagano, Tazawa	COI 3’	Mikkola & Ståhls, 2008		AM946713
D. superans	Japan, Nagano, Tazawa	COI 3’	Mikkola & Ståhls, 2008		AM946714
D. superans	Japan, Nagano, Tazawa	COI 3’	Mikkola & Ståhls, 2008		AM946715
D. superans	Japan, Nagano, Tazawa	COI 3’	Mikkola & Ståhls, 2008		AM946716
D. superans	Japan, Nagano, Tazawa	ITS2	Mikkola & Ståhls, 2008		AM946731
D. superans	Japan, Nagano, Tazawa	ITS2	Mikkola & Ståhls, 2008		AM946732
D. superans	Japan, Nagano, Tazawa	ITS2	Mikkola & Ståhls, 2008		AM946733
D. superans	Japan, Nagano, Tazawa	ITS2	Mikkola & Ståhls, 2008		AM946734
D. superans	Japan, Nagano, Tazawa	ITS2	Mikkola & Ståhls, 2008		AM946735
D. superans	Japan, Nagano, Tazawa	ITS2	Mikkola & Ståhls, 2008		AM946736
D. superans	Japan, Moriyoshi	COI 3’	Mikkola & Ståhls, 2008		AM946718
D. superans	Japan, Moriyoshi	COI 3’	Mikkola & Ståhls, 2008		AM946719
D. superans	Japan, Moriyoshi	COI 3’	Mikkola & Ståhls, 2008		AM946720
D. superans	Japan, Moriyoshi	ITS2	Mikkola & Ståhls, 2008		AM946738
D. superans	Japan, Moriyoshi	ITS2	Mikkola & Ståhls, 2008		AM946739
D. superans	Japan, Moriyoshi	ITS2	Mikkola & Ståhls, 2008		AM946740
D. superans	Japan, Kawabe	COI 3’	Mikkola & Ståhls, 2008		AM946717
D. superans	Japan, Kawabe	ITS2	Mikkola & Ståhls, 2008		AM946737
D. superans	Japan	COI 3’	Mikkola & Ståhls, 2008		AM946721
D. superans	China, Keshenketeng, Neimenggu	COI 5’	Dai et al., 2012		JN602811
D. superans	China, Keshenketeng, Neimenggu	COI 5’	Dai et al., 2012		JN602812
D. superans	China, Keshenketeng, Neimenggu	COI 5’	Dai et al., 2012		JN602813
D. superans	China, Keshenketeng, Neimenggu	COI 5’	Dai et al., 2012		JN602814
D. superans	China, Keshenketeng, Neimenggu	COI 5’	Dai et al., 2012		JN602815
D. superans	China, Keshenketeng, Neimenggu	COI 5’	Dai et al., 2012		JN602816
D. superans	China, Keshenketeng, Neimenggu	COI 5’	Dai et al., 2012		JN602817
D. superans	China, Keshenketeng, Neimenggu	COI 5’	Dai et al., 2012		JN602818
D. superans	China, Keshenketeng, Neimenggu	COI 5’	Dai et al., 2012		JN602819
D. superans	China, Keshenketeng, Neimenggu	COI 5’	Dai et al., 2012		JN602820
D. superans	China	COI 5’	Jia et al., 2011		JF826848
D. superans	China	COI 5’	Jia et al., 2011		JF826849
D. superans	China	COI 5’	Jia et al., 2011		JF826850
D. superans	China	COI 5’	Jia et al., 2011		JF826851
D. superans	China, Keshenketeng, Neimenggu	ITS2	Dai et al., 2012		JN602979
D. superans	China, Keshenketeng, Neimenggu	ITS2	Dai et al., 2012		JN602980
D. superans	China, Keshenketeng, Neimenggu	ITS2	Dai et al., 2012		JN602981
D. superans	China, Keshenketeng, Neimenggu	ITS2	Dai et al., 2012		JN602982
D. superans	China, Keshenketeng, Neimenggu	ITS2	Dai et al., 2012		JN602983
D. superans	China, Keshenketeng, Neimenggu	ITS2	Dai et al., 2012		JN602984
D. superans	China, Keshenketeng, Neimenggu	ITS2	Dai et al., 2012		JN602985
D. superans	China, Keshenketeng, Neimenggu	ITS2	Dai et al., 2012		JN602986
D. superans	China, Keshenketeng, Neimenggu	ITS2	Dai et al., 2012		JN602987
D. superans	Russia, Khabarovsk, Khekhtsir	COI 5’	the present work	DS21	KJ007778
D. superans	Russia, Khabarovsk, Khekhtsir	COI 5’	the present work	DS22	KJ007779
D. superans	Russia, Khabarovsk, Khekhtsir	COI 5’	the present work	DS23	KJ007780
D. superans	Russia, Khabarovsk, Khekhtsir	COI 5’	the present work	DS24	KJ007781
D. superans	Russia, Khabarovsk, Khekhtsir	COI 3’	the present work	DS21	KJ007778
D. superans	Russia, Khabarovsk, Khekhtsir	COI 3’	the present work	DS22	KJ007779
D. superans	Russia, Khabarovsk, Khekhtsir	COI 3’	the present work	DS23	KJ007780
D. superans	Russia, Khabarovsk, Khekhtsir	COI 3’	the present work	DS24	KJ007781
D. superans	Russia, Khabarovsk, Khekhtsir	ITS2	the present work	DS21	KJ007753
D. superans	Russia, Khabarovsk, Khekhtsir	ITS2	the present work	DS22	KJ007754
D. superans	Russia, Khabarovsk, Khekhtsir	ITS2	the present work	DS23	KJ007755
D. superans	Russia, Khabarovsk, Khekhtsir	ITS2	the present work	DS24	KJ007756
D. superans	Russia, Khabarovsk, Khekhtsir	COII	the present work	DS21	KJ007804

D. superans	Russia, Khabarovsk, Khekhtsir	COII	the present work	DS22	KJ007805
D. superans	Russia, Khabarovsk, Khekhtsir	COII	the present work	DS23	KJ007806
D. superans	Russia, Khabarovsk, Khekhtsir	COII	the present work	DS24	KJ007807
D. spectabilis	Japan, Nagano	ITS2	Mikkola & Ståhls, 2008		AM946722
D. spectabilis	Japan, Nagano	ITS2	Mikkola & Ståhls, 2008		AM946743
D. spectabilis	Japan, Shimotogawa	ITS2	Mikkola & Ståhls, 2008		AM946741
D. spectabilis	Japan, Shimotogawa	ITS2	Mikkola & Ståhls, 2008		AM946742
D. spectabilis	China	COI 5’	Dai et al., 2012		JN602785
D. spectabilis	China	COI 5’	Dai et al., 2012		JN602786
D. spectabilis	China	COI 5’	Dai et al., 2012		JN602787
D. spectabilis	China	COI 5’	Dai et al., 2012		JN602788
D. spectabilis	China	COI 5’	Dai et al., 2012		JN602789
D. spectabilis	China	COI 5’	Dai et al., 2012		JN602790
D. spectabilis	China	COI 5’	Dai et al., 2012		JN602791
D. spectabilis	China	COI 5’	Dai et al., 2012		JN602792
D. spectabilis	China	COI 5’	Dai et al., 2012		JN602793
D. spectabilis	China	COI 5’	Dai et al., 2012		JN602794
D. spectabilis	China	COI 5’	Dai et al., 2012		JN602795
D. spectabilis	China	COI 5’	Dai et al., 2012		JN602796
D. spectabilis	China	COI 5’	Dai et al., 2012		JN602797
D. spectabilis	China	COI 5’	Dai et al., 2012		JN602798
D. spectabilis	China	ITS2	Dai et al., 2012		JN602969
D. spectabilis	China	ITS2	Dai et al., 2012		JN602970
D. spectabilis	China	ITS2	Dai et al., 2012		JN602971
D. spectabilis	China	ITS2	Dai et al., 2012		JN602972
D. spectabilis	China	ITS2	Dai et al., 2012		JN602973
D. spectabilis	China	ITS2	Dai et al., 2012		JN602974
D. spectabilis	China	ITS2	Dai et al., 2012		JN602975
D. spectabilis	China	ITS2	Dai et al., 2012		JN602976
D. spectabilis	China	ITS2	Dai et al., 2012		JN602977
D. spectabilis	China	ITS2	Dai et al., 2012		JN602978
D. spectabilis	South Korea	COI 5’	Dai et al., 2012		JN087390
D. spectabilis	South Korea	COI 5’	Dai et al., 2012		KC135936
D. punctatus	China	COI 5’	Dai et al., 2012		JF826793
D. punctatus	China	COI 5’	Dai et al., 2012		JF826794
D. punctatus	China	COI 5’	Dai et al., 2012		JF826795
D. punctatus	China	COI 5’	Dai et al., 2012		JF826796
D. punctatus	China	COI 5’	Dai et al., 2012		JF826797
D. punctatus	China	COI 5’	Dai et al., 2012		JF826798
D. punctatus	China	COI 5’	Dai et al., 2012		JF826799
D. punctatus	China	COI 5’	Dai et al., 2012		JF826800
D. punctatus	China	COI 5’	Dai et al., 2012		JF826801
D. punctatus	China	COI 5’	Dai et al., 2012		JF826802
D. punctatus	China	COI 5’	Dai et al., 2012		JF826803
D. punctatus	China	COI 5’	Dai et al., 2012		JF826804
D. punctatus	China	COI 5’	Dai et al., 2012		JF826805
D. punctatus	China	COI 5’	Dai et al., 2012		JF826806
D. punctatus	China	COI 5’	Dai et al., 2012		JF826807
D. punctatus	China	COI 5’	Dai et al., 2012		JF826808
D. punctatus	China	COI 5’	Dai et al., 2012		JF826809
D. punctatus	China	COI 5’	Dai et al., 2012		JF826810
D. punctatus	China	COI 5’	Dai et al., 2012		JF826811
D. punctatus	China	COI 5’	Dai et al., 2012		JF826812
D. punctatus	China	COI 5’	Dai et al., 2012		JF826813
D. punctatus	China	COI 5’	Dai et al., 2012		JF826814
D. punctatus	China	COI 5’	Dai et al., 2012		JF826815
D. punctatus	China	COI 5’	Dai et al., 2012		JF826816
D. punctatus	China	COI 5’	Dai et al., 2012		JF826817
D. punctatus	China	COI 5’	Dai et al., 2012		JF826818
D. punctatus	China	COI 5’	Dai et al., 2012		JF826819
D. punctatus	China	COI 5’	Dai et al., 2012		JF826820
D. punctatus	China	COI 5’	Dai et al., 2012		JF826821
D. punctatus	China	COI 5’	Dai et al., 2012		JF826823
D. punctatus	China	COI 5’	Dai et al., 2012		JF826824
D. punctatus	China	COI 5’	Dai et al., 2012		JF826825
D. punctatus	China	COI 5’	Dai et al., 2012		JF826826
D. punctatus	China	COI 5’	Dai et al., 2012		JF826827
D. punctatus	China	COI 5’	Dai et al., 2012		JF826828
D. punctatus	China	COI 5’	Dai et al., 2012		JF826829
D. punctatus	China	COI 5’	Dai et al., 2012		JF826830
D. punctatus	China	COI 5’	Dai et al., 2012		JF826831

D. punctatus	China	COI 5’	Dai et al., 2012	JF826832
D. punctatus	China	COI 5’	Dai et al., 2012	JF826833
D. punctatus	China	ITS2	Dai et al., 2012	JN602948
D. punctatus	China	ITS2	Dai et al., 2012	JN602949
D. punctatus	China	ITS2	Dai et al., 2012	JN602950
D. punctatus	China	ITS2	Dai et al., 2012	JN602951
D. punctatus	China	ITS2	Dai et al., 2012	JN602952
D. punctatus	China	ITS2	Dai et al., 2012	JN602953
D. punctatus	China	ITS2	Dai et al., 2012	JN602954
D. punctatus	China	ITS2	Dai et al., 2012	JN602955
D. punctatus	China	ITS2	Dai et al., 2012	JN602956
D. punctatus	China	ITS2	Dai et al., 2012	JN602957
D. punctatus	China	ITS2	Dai et al., 2012	JN602958
D. punctatus	China	ITS2	Dai et al., 2012	JN602959
D. punctatus	China	ITS2	Dai et al., 2012	JN602960
D. punctatus	China	ITS2	Dai et al., 2012	JN602961
D. punctatus	China	ITS2	Dai et al., 2012	JN602962
D. punctatus	China	ITS2	Dai et al., 2012	JN602963
D. punctatus	China	ITS2	Dai et al., 2012	JN602964
D. punctatus	China	ITS2	Dai et al., 2012	JN602965
D. punctatus	China	ITS2	Dai et al., 2012	JN602966
D. punctatus	China	ITS2	Dai et al., 2012	JN602967
D. punctatus	China	ITS2	Dai et al., 2012	JN602968
D. punctatus	China	ITS2	Dai et al., 2012	JN603004
D. punctatus	China	ITS2	Dai et al., 2012	JN603005
D. punctatus	China	ITS2	Dai et al., 2012	JN603006
D. punctatus	China	ITS2	Jia et al., 2011	AJ577260
D. tabulaeformis	China, Hebei	COI 5’	Dai et al., 2012	JN602844
D. tabulaeformis	China, Hebei	COI 5’	Dai et al., 2012	JN602845
D. tabulaeformis	China, Hebei	COI 5’	Dai et al., 2012	JN602846
D. tabulaeformis	China, Hebei	COI 5’	Dai et al., 2012	JN602847
D. tabulaeformis	China, Hebei	COI 5’	Dai et al., 2012	JN602848
D. tabulaeformis	China, Hebei	COI 5’	Dai et al., 2012	JN602849
D. tabulaeformis	China, Hebei	COI 5’	Dai et al., 2012	JN602850
D. tabulaeformis	China, Hebei	COI 5’	Dai et al., 2012	JN602851
D. tabulaeformis	China, Hebei	COI 5’	Dai et al., 2012	JN602852
D. tabulaeformis	China, Hebei	COI 5’	Dai et al., 2012	JN602853
D. tabulaeformis	China, Hebei	COI 5’	Dai et al., 2012	JN602854
D. tabulaeformis	China, Hebei	COI 5’	Dai et al., 2012	JN602855
D. tabulaeformis	China, Hebei	COI 5’	Dai et al., 2012	JN602856
D. tabulaeformis	China, Hebei	COI 5’	Dai et al., 2012	JN602857
D. tabulaeformis	China, Hebei	COI 5’	Dai et al., 2012	JN602858
D. tabulaeformis	China, Hebei	COI 5’	Dai et al., 2012	JN602859
D. tabulaeformis	China, Hebei	COI 5’	Dai et al., 2012	JN602860
D. tabulaeformis	China, Hebei	COI 5’	Dai et al., 2012	JN602861
D. tabulaeformis	China, Hebei	COI 5’	Dai et al., 2012	JN602862
D. tabulaeformis	China, Hebei	COI 5’	Dai et al., 2012	JN602863
D. tabulaeformis	China, Hebei	COI 5’	Dai et al., 2012	JN602864
D. tabulaeformis	China, Hebei	COI 5’	Dai et al., 2012	JN602865
D. tabulaeformis	China, Hebei	COI 5’	Dai et al., 2012	JN602866
D. tabulaeformis	China, Hebei	COI 5’	Dai et al., 2012	JN602867
D. tabulaeformis	China, Hebei	COI 5’	Dai et al., 2012	JN602868
D. tabulaeformis	China, Hebei	COI 5’	Dai et al., 2012	JN602869
D. tabulaeformis	China, Hebei	COI 5’	Dai et al., 2012	JN602870
D. tabulaeformis	China, Hebei	COI 5’	Dai et al., 2012	JN602871
D. tabulaeformis	China, Hebei	COI 5’	Dai et al., 2012	JN602872
D. tabulaeformis	China, Hebei	COI 5’	Dai et al., 2012	JN602873
D. tabulaeformis	China, Hebei	COI 5’	Dai et al., 2012	JN602874
D. tabulaeformis	China, Hebei	COI 5’	Dai et al., 2012	JN602875
D. tabulaeformis	China, Hebei	COI 5’	Dai et al., 2012	JN602876
D. tabulaeformis	China, Hebei	COI 5’	Dai et al., 2012	JN602877
D. tabulaeformis	China, Hebei	COI 5’	Dai et al., 2012	JN602878
D. tabulaeformis	China, Hebei	COI 5’	Jia et al., 2011	JF826834
D. tabulaeformis	China, Hebei	COI 5’	Jia et al., 2011	JF826835
D. tabulaeformis	China, Hebei	COI 5’	Jia et al., 2011	JF826836
D. tabulaeformis	China, Hebei	COI 5’	Jia et al., 2011	JF826837
D. tabulaeformis	China, Hebei	COI 5’	Jia et al., 2011	JF826838
D. tabulaeformis	China, Hebei	COI 5’	Jia et al., 2011	JF826839
D. tabulaeformis	China, Hebei	ITS2	Dai et al., 2012	JN603018
D. tabulaeformis	China, Hebei	ITS2	Dai et al., 2012	JN603019
D. tabulaeformis	China, Hebei	ITS2	Dai et al., 2012	JN603020

D. tabulaeformis	China, Hebei	ITS2	Dai et al., 2012	JN603021
D. tabulaeformis	China, Hebei	ITS2	Dai et al., 2012	JN603022
D. tabulaeformis	China, Hebei	ITS2	Dai et al., 2012	JN603023
D. tabulaeformis	China, Hebei	ITS2	Dai et al., 2012	JN603024
D. tabulaeformis	China, Hebei	ITS2	Dai et al., 2012	JN603025
D. tabulaeformis	China, Hebei	ITS2	Dai et al., 2012	JN603026
D. tabulaeformis	China, Hebei	ITS2	Dai et al., 2012	JN603027
D. tabulaeformis	China, Hebei	ITS2	Dai et al., 2012	JN603028
D. tabulaeformis	China, Hebei	ITS2	Dai et al., 2012	JN603029
D. tabulaeformis	China, Hebei	ITS2	Dai et al., 2012	JN603030
D. tabulaeformis	China, Hebei	ITS2	Dai et al., 2012	JN603031
D. tabulaeformis	China, Hebei	ITS2	Dai et al., 2012	JN603032
D. tabulaeformis	China, Hebei	ITS2	Dai et al., 2012	JN603033
D. tabulaeformis	China, Hebei	ITS2	Dai et al., 2012	JN603034
D. tabulaeformis	China, Hebei	ITS2	Dai et al., 2012	JN603035
D. tabulaeformis	China, Hebei	ITS2	Dai et al., 2012	JN603036
D. tabulaeformis	China, Hebei	ITS2	Dai et al., 2012	JN603037
D. tabulaeformis	China, Hebei	ITS2	Dai et al., 2012	JN603038
D. tabulaeformis	China, Hebei	ITS2	Dai et al., 2012	JN603039
D. tabulaeformis	China, Hebei	ITS2	Dai et al., 2012	JN603040
D. tabulaeformis	China, Hebei	ITS2	Dai et al., 2012	JN603041
D. tabulaeformis	China, Hebei	ITS2	Dai et al., 2012	JN603042
D. tabulaeformis	China, Hebei	ITS2	Dai et al., 2012	JN603043
D. tabulaeformis	China, Hebei	ITS2	Dai et al., 2012	JN603044
D. houi	China, Yunnan	COI 5’	Dai et al., 2012	JN602799
D. houi	China, Yunnan	COI 5’	Dai et al., 2012	JN602800
D. houi	China, Yunnan	COI 5’	Dai et al., 2012	JN602801
D. houi	China, Yunnan	COI 5’	Dai et al., 2012	JN602802
D. houi	China, Yunnan	COI 5’	Dai et al., 2012	JN602803
D. houi	China, Yunnan	COI 5’	Dai et al., 2012	JN602804
D. houi	China, Yunnan	COI 5’	Dai et al., 2012	JN602805
D. houi	China, Yunnan	COI 5’	Dai et al., 2012	JN602806
D. houi	China, Yunnan	COI 5’	Dai et al., 2012	JN602807
D. houi	China, Yunnan	COI 5’	Dai et al., 2012	JN602808
D. houi	China, Yunnan	COI 5’	Dai et al., 2012	JN602809
D. houi	China, Yunnan	COI 5’	Dai et al., 2012	JN602810
D. houi	China, Yunnan	COI 5’	Jia et al., 2011	JF826861
D. houi	China, Yunnan	COI 5’	Jia et al., 2011	JF826862
D. houi	China, Yunnan	COI 3’	Leng, 2010	HM071844
D. houi	China, Yunnan	COI 3’	Leng, 2010	HM071845
D. houi	China, Yunnan	ITS2	Dai et al., 2012	JN603007
D. houi	China, Yunnan	ITS2	Dai et al., 2012	JN603008
D. houi	China, Yunnan	ITS2	Dai et al., 2012	JN603009
D. houi	China, Yunnan	ITS2	Dai et al., 2012	JN603010
D. houi	China, Yunnan	ITS2	Dai et al., 2012	JN603011
D. houi	China, Yunnan	ITS2	Dai et al., 2012	JN603012
D. houi	China, Yunnan	ITS2	Dai et al., 2012	JN603013
D. houi	China, Yunnan	ITS2	Dai et al., 2012	JN603014
D. houi	China, Yunnan	ITS2	Dai et al., 2012	JN603015
D. houi	China, Yunnan	ITS2	Dai et al., 2012	JN603016
D. houi	China, Yunnan	ITS2	Dai et al., 2012	JN603017
D. houi	China, Yunnan	COII	Leng, 2010	HM104580
D. houi	China, Yunnan	COII	Leng, 2010	HM104581
D. houi	China, Yunnan	COII	Leng, 2010	HM104587
D. houi	China, Yunnan	COII	Leng, 2010	HM104588
D. kikuchii	China, Yunnan	COI 5’	Dai et al., 2012	JN602821
D. kikuchii	China, Yunnan	COI 5’	Dai et al., 2012	JN602822
D. kikuchii	China, Yunnan	COI 5’	Dai et al., 2012	JN602823
D. kikuchii	China, Yunnan	COI 5’	Dai et al., 2012	JN602824
D. kikuchii	China, Yunnan	COI 5’	Dai et al., 2012	JN602825
D. kikuchii	China, Yunnan	COI 5’	Dai et al., 2012	JN602826
D. kikuchii	China, Yunnan	COI 5’	Dai et al., 2012	JN602827
D. kikuchii	China, Yunnan	COI 5’	Dai et al., 2012	JN602828
D. kikuchii	China, Yunnan	COI 5’	Dai et al., 2012	JN602829
D. kikuchii	China, Yunnan	COI 5’	Dai et al., 2012	JN602830
D. kikuchii	China, Yunnan	COI 5’	Dai et al., 2012	JN602831
D. kikuchii	China, Yunnan	COI 5’	Dai et al., 2012	JN602832
D. kikuchii	China, Yunnan	COI 5’	Dai et al., 2012	JN602833
D. kikuchii	China, Yunnan	COI 5’	Dai et al., 2012	JN602834
D. kikuchii	China, Yunnan	COI 5’	Dai et al., 2012	JN602835
D. kikuchii	China, Yunnan	COI 5’	Dai et al., 2012	JN602836

D. kikuchii	China, Yunnan	COI 5’	Dai et al., 2012	JN602837
D. kikuchii	China, Yunnan	COI 5’	Dai et al., 2012	JN602838
D. kikuchii	China, Yunnan	COI 5’	Dai et al., 2012	JN602839
D. kikuchii	China, Yunnan	COI 5’	Dai et al., 2012	JN602840
D. kikuchii	China, Yunnan	COI 5’	Dai et al., 2012	JN602841
D. kikuchii	China, Yunnan	COI 5’	Dai et al., 2012	JN602842
D. kikuchii	China, Yunnan	COI 5’	Dai et al., 2012	JN602843
D. kikuchii	China, Yunnan	COI 5’	Jia et al., 2011	JF826852
D. kikuchii	China, Yunnan	COI 5’	Jia et al., 2011	JF826853
D. kikuchii	China, Yunnan	COI 5’	Jia et al., 2011	JF826854
D. kikuchii	China, Yunnan	COI 5’	Jia et al., 2011	JF826855
D. kikuchii	China, Yunnan	COI 5’	Jia et al., 2011	JF826856
D. kikuchii	China, Yunnan	COI 5’	Jia et al., 2011	JF826857
D. kikuchii	China, Yunnan	COI 5’	Jia et al., 2011	JF826858
D. kikuchii	China, Yunnan	COI 5’	Jia et al., 2011	JF826859
D. kikuchii	China, Yunnan	COI 5’	Jia et al., 2011	JF826860
D. kikuchii	China, Yunnan	ITS2	Dai et al., 2012	JN602988
D. kikuchii	China, Yunnan	ITS2	Dai et al., 2012	JN602989
D. kikuchii	China, Yunnan	ITS2	Dai et al., 2012	JN602990
D. kikuchii	China, Yunnan	ITS2	Dai et al., 2012	JN602991
D. kikuchii	China, Yunnan	ITS2	Dai et al., 2012	JN602992
D. kikuchii	China, Yunnan	ITS2	Dai et al., 2012	JN602993
D. kikuchii	China, Yunnan	ITS2	Dai et al., 2012	JN602994
D. kikuchii	China, Yunnan	ITS2	Dai et al., 2012	JN602995
D. kikuchii	China, Yunnan	ITS2	Dai et al., 2012	JN602996
D. kikuchii	China, Yunnan	ITS2	Dai et al., 2012	JN602997
D. kikuchii	China, Yunnan	ITS2	Dai et al., 2012	JN602998
D. kikuchii	China, Yunnan	ITS2	Dai et al., 2012	JN602999
D. kikuchii	China, Yunnan	ITS2	Dai et al., 2012	JN603000
D. kikuchii	China, Yunnan	ITS2	Dai et al., 2012	JN603001
D. kikuchii	China, Yunnan	ITS2	Dai et al., 2012	JN603002
D. kikuchii	China, Yunnan	ITS2	Dai et al., 2012	JN603003
D. kikuchii	China, Yunnan	COII	Leng, 2010	HM104582
D. kikuchii	China, Yunnan	COII	Leng, 2010	HM104583
D. kikuchii	China, Yunnan	COII	Leng, 2010	HM104584
D. kikuchii	China, Yunnan	COII	Leng, 2010	HM104585
